# Overexpression of primary microRNA 221/222 in acute myeloid leukemia

**DOI:** 10.1186/1471-2407-13-364

**Published:** 2013-07-29

**Authors:** Anna Rommer, Katarina Steinleitner, Hubert Hackl, Christine Schneckenleithner, Maria Engelmann, Marcel Scheideler, Irena Vlatkovic, Robert Kralovics, Sabine Cerny-Reiterer, Peter Valent, Heinz Sill, Rotraud Wieser

**Affiliations:** 1 Department of Medicine I, Medical University of Vienna, Währinger Gürtel 18-20, 1090 Vienna, Austria; 2 Comprehensive Cancer Center of the Medical University of Vienna, Vienna, Austria; 3 Biocenter, Division of Bioinformatics, Innsbruck Medical University, Innrain 80, 6020 Innsbruck, Austria; 4 Present address: Institute of Pharmacology and Toxicology, University of Veterinary Medicine, Vienna, Austria; 5 Institute for Genomics and Bioinformatics, Graz University of Technology, Petersgasse 14, 8010 Graz, Austria; 6 Center for Molecular Medicine of the Austrian Academy of Sciences, Lazarettgasse 14, Vienna, Austria; 7 Division of Hematology and Hemostaseology, Department of Medicine I, Medical University of Vienna, Währinger Gürtel 18-20, 1090 Vienna, Austria; 8 Ludwig Boltzmann Cluster Oncology, Währinger Gürtel 18-20, 1090 Vienna, Austria; 9 Division of Hematology, Medical University of Graz, Auenbruggerplatz 38, 8036 Graz, Austria

**Keywords:** AML, miR-221, pri-miRNA, lncRNA

## Abstract

**Background:**

Acute myeloid leukemia (AML) is a hematopoietic malignancy with a dismal outcome in the majority of cases. A detailed understanding of the genetic alterations and gene expression changes that contribute to its pathogenesis is important to improve prognostication, disease monitoring, and therapy. In this context, leukemia-associated misexpression of microRNAs (miRNAs) has been studied, but no coherent picture has emerged yet, thus warranting further investigations.

**Methods:**

The expression of 636 human miRNAs was compared between samples from 52 patients with AML and 13 healthy individuals by highly specific locked nucleic acid (LNA) based microarray technology. The levels of individual mature miRNAs and of primary miRNAs (pri-miRs) were determined by quantitative reverse transcriptase (qRT) PCR. Transfections and infections of human cell lines were performed using standard procedures.

**Results:**

64 miRNAs were significantly differentially expressed between AML and controls. Further studies on the clustered miRNAs 221 and 222, already known to act as oncogenes in other tumor types, revealed a deficiency of human myeloid cell lines to process vector derived precursor transcripts. Moreover, endogenous pri-miR-221/222 was overexpressed to a substantially higher extent than its mature products in most primary AML samples, indicating that its transcription was enhanced, but processing was rate limiting, in these cells. Comparison of samples from the times of diagnosis, remission, and relapse of AML demonstrated that pri-miR-221/222 levels faithfully reflected the stage of disease.

**Conclusions:**

Expression of some miRNAs is strongly regulated at the posttranscriptional level in AML. Pri-miR-221/222 represents a novel molecular marker and putative oncogene in this disease.

## Background

Acute myeloid leukemia (AML) is a frequently fatal malignant disease of hematopoietic stem and progenitor cells (HSPCs). Prognostic factors include patient age, antecedent hematological disease, preceding cytotoxic treatments for a primary disorder, and the presence of specific cytogenetic, molecular, and epigenetic aberrations [1-7]. Identification and investigation of such somatic genetic alterations has enhanced our understanding of disease biology, augments diagnosis and prognostication, and may aid monitoring of the course of disease [1,3,7,8]. Although cytogenetics and molecular genetics have already facilitated great progress in these respects, novel technologies like next generation sequencing and biological breakthroughs like the identification of microRNAs (miRNAs) and long noncoding RNAs (lncRNAs) as entirely novel gene classes still provide fundamental additional insights. In this context, the expression, functions, and potential prognostic value of miRNAs in AML have been studied by a number of research groups over the past few years [9-15]. In contrast, little is known about the expression and roles of lncRNAs in this disease.

miRNAs are small (~22 nucleotide, nt) RNA molecules which typically regulate multiple target genes at the levels of mRNA stability and translation efficiency [16,17]. They are excised through sequential processing steps from larger, usually polymerase II transcribed, RNA molecules termed primary miRNAs (pri-miRNAs). One pri-miRNA molecule may give rise to one or several miRNA species, with the respective genomic region referred to as a miRNA cluster in the latter case [18]. Classical pri-miRNA processing consists of two endonucleolytic cleavage steps: the first one is carried out in the nucleus by the RNase Drosha and gives rise to ~70 nt long, hairpin shaped precursor (pre-) miRNA molecules. These are exported to the cytoplasm and further processed by another RNase, Dicer, to yield mature miRNAs [18-21]. After incorporation into the RNA induced silencing complex (RISC), miRNAs recognize their target mRNAs through a 7–8 nt seed sequence and repress their translation and stability [16,17,20,21]. In addition to this well described way of action of mature miRNAs, biochemical and biological functions have recently been ascribed to some pri-miRNAs [22,23], thus greatly expanding the potential biological relevance of miRNA genes.

Large scale expression analyses of mature miRNAs revealed specific miRNA patterns that were associated with recurrent genetic abnormalities in AML [10-12,24]. Certain miRNAs were deregulated in AML compared to healthy bone marrow (BM), peripheral blood (PB), or CD34 positive (CD34+) HSPCs [10-12,24,25], and miRNA signatures predictive of survival have been identified [9,10]. However, results from different research groups exhibited only limited concordance, most likely due to differences between patient collectives, types of healthy control, and methodologies [13].

In this study, we re-addressed the question which miRNAs are differentially expressed between AML and healthy controls, and therefore might contribute to leukemogenesis. Using highly specific locked nucleic acid (LNA) oligonucleotide arrays capable of detecting 559 annotated and 77 proprietary human miRNAs, 64 miRNAs were found to be significantly misexpressed in AML. Further studies on the clustered miRNAs 221 and 222 revealed that pri-miR221/222 was overexpressed to a much higher extent than its mature products, which is best explained by an increased rate of transcription on the background of a limited processing capacity. Because pri-miR-221/222 is strongly overexpressed in a large proportion of patients with AML, it may represent a novel molecular marker and a putative oncogene in this disease.

## Methods

### Primary samples from patients and controls

This study was approved by the Ethics Committee of the Medical University of Vienna (EK no 609/2011). In some cases, archival samples were used. Written informed consent was obtained prior to sample collection from all other subjects. Data were analyzed anonymously.

For miRNA microarray hybridization, 52 AML PB samples and 13 control samples were used (Additional file 1: Tables S1A and B). The AML samples had been collected at the time of diagnosis, strongly enriched for leukemic blasts using Ficoll, and vitally frozen. Of the healthy control samples, three were CD34+ HPSCs enriched from bone marrow (BM) and five were BM mononuclear cells (MNCs) (all purchased from Lonza, Basel, Switzerland). Another five were PB samples from healthy volunteers, and were subjected to erythrocyte lysis prior to RNA isolation (Additional file 1: Table S1A). Twenty-two and 9 of these AML and control samples, respectively, were also used for qRT-PCR for mature miR-221 and 222. Characteristics of 27 additional diagnostic AML samples used to determine the ratio between pri-miR-221/222 and mature miR-221, as well as the expression of pri-miR-221/222 and other pri-miRNAs are summarized in Additional file 1: Table S1C. Eight additional control samples used for qRT-PCR experiments are included in Additional file 1: Table S1A. To investigate pri-miR-221/222 expression during the course of disease, four paired samples from the time of diagnosis and remission and three paired samples from the time of diagnosis and relapse were analyzed (Additional file 1: Table S1D).

### Production and hybridization of miRNA microarrays, and primary data analysis

miRNA microarrays were produced as previously described [26,27]. Briefly, LNA modified oligonucleotide probes specific for the 559 human miRNAs contained in miRBase version 9.2 (http://www.mirbase.org/) as well as probes for 77 proprietary miRPlus sequences (Exiqon, Vedbaek, Denmark) were spotted onto Hisens epoxy-coated glass slides (Schott Nexterion, Louisville, KY, USA) in eight replicates.

RNA was extracted with Trizol (Life Technologies, Carlsbad, CA, USA), subjected to quality control using a Bioanalyzer 2100 (Agilent Technologies, Santa Clara, CA, USA), and labelled with Hy3 using the miRCURY™ LNA microRNA array labelling kit (Exiqon). A Hy5 labelled mix of all samples was included in each hybridization as a common reference. Arrays were hybridized overnight at 60°C in microarray hybridization chambers (Corning, Corning, NY, USA). Hybridization and wash buffers from the miRCURY LNA microRNA Array Kit (Exiqon) were used according to the manufacturer’s instructions. Arrays were scanned with a GenePix 4100A Microarray Scanner and evaluated with GenpixPro 5.1 software (Molecular Devices, Sunnyvale, CA, USA). Primary data analysis was performed using ArrayNorm [28]. Features were filtered for low quality spots, the local background was subtracted, spots were normalized to the global mean, and the ratios between the sample of interest and the common reference were log2 transformed.

### Cell lines, miRNA expression vectors, stable transfections, and infections

KG1 and KG1a cells [29] were obtained from the German Collection of Microorganisms and Cell Cultures, Braunschweig, Germany. HNT34 [30] and MPD [31] cells were kindly provided by Dr. Hiroyuki Hamaguchi, Musashino Red Cross Hospital, Tokyo, Japan, and Dr. Cassandra Paul, Wright State University, Dayton. Ohio, USA, respectively. HL60 [32], HEL [33], U937 [34], HeLa [35], MCF7 [36], and 293 T [37] cells were obtained from cell culture collections of the Medical University of Vienna. All cell lines were regularly tested for mycoplasma contamination.

The human myeloid cell lines KG1, HL60, HEL, U937, MPD, and HNT34 were cultured in Roswell Park Memorial Institute (RPMI) 1640 medium supplemented with 10%Fetal Bovine Serum (FBS) and 1%Penicillin-Streptomycin-Glutamine (PSG; all from Life Technologies, Carlsbad, CA, USA) in a humidified incubator at 37°C and 5%CO_2_. The same media were used for KG1a cells, except that they contained 20%FBS. The adherent cell lines HeLa, MCF7, and 293 T were maintained in Dulbecco’s Modified Eagle Medium (DMEM; Life Technologies) with 10%FBS and 1%PSG.

Plasmid miR-Vec-221/222, which contains the miR-221/222 cluster along with a blasticidin resistence gene in the pMSCV backbone, as well as the corresponding empty vector (miR-Vec) were kindly provided by the Agami lab [38]. They were stably transfected into HL60 cells by electroporation and transfectants were selected using 2 μg/ml Blasticidin (Invivogen, San Diego, CA, USA).

pEZX-MR03-miR-221 (Homo sapiens microRNA miR-221 stem-loop expression clone, #HMIR0369), an HIV based lentiviral vector containing the miR-221 precursor, and pEZX-MR01-control (#CMIR0001-MR01), which contains a scrambled sequence instead, were obtained from GeneCopoeia (Rockville, Maryland, USA). They were infected into the myeloid cell lines HL60, KG-1, and KG-1a using standard procedures [39]. Infected cells were sorted for GFP positivity on a FACS Aria (BD Biosciences, NJ, USA).

### RNA extraction, cDNA synthesis, and quantitative reverse transcriptase PCR (qRT-PCR)

Total RNA was isolated from primary samples and cell lines using Trizol (Life Technologies). For detection of pri-miRNAs, RNA was treated with DNase I and converted to cDNA with M-MLV reverse transcriptase primed by random hexamer oligonucleotides (all reagents from Life Technologies). qRT-PCR was performed in an ABI Step One Plus sequence detection system (Applied Biosystems, Life Technologies) using the Mesa Green qPCR Master Mix Plus (Eurogentec, Liège, Belgium) and the primers listed in Additional file 2: Table S2A (synthesized by MWG Eurofins, Ebersberg, Germany). All primer pairs were subjected to standard curve analysis and yielded slopes between -3.0 and -3.5, indicating optimal or near optimal amplification efficiencies. Levels of mature miR-221 and miR-222 and of RNU6B were measured using Taqman assays (ID000524, hsa-miR-221 TaqMan Assay; ID002276, hsa-miR-222 TaqMan Assay; ID001093, mature miR control-RNU6B TaqMan Assay; Applied Biosystems). qRT-PCR reactions were performed in triplicate. The relative expression of pri-miRNAs compared to the housekeeping gene beta-2-microglobulin, and of mature miRNAs relative to RNU6B, were calculated according to the ΔΔCt method [40].

### RNA deep sequencing analysis and prediction of putative pri-miR 221/222 transcripts

RNA deep sequencing optimized for detection of lncRNAs was performed as described [41]. In short, total RNA was extracted from human Hs27 foreskin fibroblasts with TRIreagent (Sigma-Aldrich, Seelze, Germany), treated with DNaseI (DNA-free kit, Ambion, Life Technologies), depleted of ribosomal RNA using RiboMinus Transcriptome Isolation Kit Human/Mouse (Life Technologies) and Ribo-Zero rRNA Removal Kit Human/Mouse/Rat (Epicentre Biotechnologies, Madison, WI, USA), and fragmented by hydrolysis. Double stranded cDNA was generated with SuperScript II Reverse Transcriptase (Life Technologies). The RNA-Seq library was prepared using the Chip-Seq DNA Sample Prep Kit (Illumina, San Diego, CA, USA), and sequenced using an Illumina Genome Analyzer II and Illumina HiSeq 2000 systems. 36 bp and 51 bp single end reads were aligned to human genome build hg18 using Bowtie (http://bowtie-bio.sourceforge.net/index.shtml) and visualized on the University of California Santa Cruz (UCSC) genome browser (http://genome.ucsc.edu/) (Vlatkovic IM, manuscript in preparation), together with publically available global run-on sequencing (GRO-Seq) data [42], ENCODE histone modification Chip-Seq data generated by the BROAD Institute, and RefSeq Genes.

### Cloning of reporter vectors, transient transfections, and luciferase reporter assays

Two fragments surrounding the transcription start site predicted for the putative 28.2 kb pri-miR-221/222 transcript were amplified from PB leukocyte genomic DNA using the primers shown in Additional file 2: Table S2B and Phusion High Fidelity Polymerase (New England Biolabs, Ipswich, MA, USA). The resulting PCR products were ligated into the pGL3-Promotor (pGL3-P) vector (Promega, Madison, WI, USA) using the SacI and XhoI restriction sites engineered onto the PCR primers to generate reporter vectors pGL3-P(-1874/+45) and pGL3-P(+17/+1952).

Subconfluent cultures of 293 T, HeLa and MCF7 cells growing in 24-well plates were transfected with 1 μg of the indicated pGL3-P vector derivative and 30 ng of the renilla luciferase vector pGL 4.70 (Promega, Madison, WI, USA), using JetPei Transfection Reagent (Polyplus Transfections, Illkirch, France) according to the manufacturer’s instructions. 48 h later, luciferase activities were measured using a Tristar LB941 (Berthold Technologies, Bad Wildbad, Germany) and the Dual-Luciferase Reporter Assay (Promega, Madison, WI, USA). Firefly luciferase activity was normalized to Renilla luciferase activity to control for transfection efficiency.

### Statistical analyses

For statistical analyses of miRNA microarray data, only features with a valid human miRNA annotation, and which were detectable in at least half of the relevant samples (i.e., 27, 2, 3, and 3 of the AML, control CD34+, control BM, and control PB samples, respectively) were considered. One-way ANOVA was performed to test if a miRNA was differentially expressed in one of the classes, and a moderated t-test (R/Biconductor package limma) was used to test if a miRNA was differentially expressed between two respective classes. Resulting p-values were adjusted for multiple hypothesis testing based on the false discovery rate (FDR) [43].

Spearman’s rank correlation was used to determine associations between miRNA expression as a continuous variable and the clinical parameters age, white blood cell count, percentage of blasts, lactate dehydrogenase (LDH), and cytogenetic risk. The point biserial correlation coefficient was used to show associations between miRNA expression and the dichotomous parameters gender and achievement of complete remission (CR), and significance was probed using the Wilcoxon rank-sum test. Associations between miRNA expression levels and the FAB type were given by the eta coefficient and significance was calculated by one-way ANOVA. All p-values were adjusted for multiple hypothesis testing based on the FDR [43]. All calculations were performed using R.

For experiments using cultured cells at least three independent biological replicates were performed. Results are expressed as means ± standard error of the mean (SEM), or, where indicated, one representative experiment with standard deviations (SDs) from technical replicates is shown. Student’s two-tailed t-test at a significance level of 0.05 was employed to probe differences between groups for statistical significance.

## Results

### Microarray analyses identify miRNAs deregulated in AML compared to healthy controls

To compare miRNA expression profiles between AML and normal controls, RNA from 52 AML samples (Additional file 1: Table S1B; enriched for leukemic blasts through Ficoll purification) and 13 healthy donors (5 peripheral blood (PB), 5 bone marrow (BM), and 3 BM CD34+; Additional file 1: Table S1A) was hybridized to microarrays containing LNA probes specifically detecting 559 human miRNAs deposited in miRBase 9.2 as well as 77 proprietary miRPlus sequences. Only miRNAs that were expressed in more than half of each the AML, healthy PB, healthy BM, and healthy CD34+ samples were considered for the respective comparisons. At a false discovery rate (FDR) of <5%, 3 miRNAs were up- and 10 downregulated in AML versus control CD34+ cells. 23 miRNAs were up- and 22 downregulated in AML compared to healthy BM, and 17 miRNAs were expressed at higher and 19 at lower levels in AML than in normal PB. 22 of these miRNAs were deregulated compared to two out of the three control tissues, and only four showed significantly altered expression with respect to all three types of control (Additional file 3: Table S3).

miRNA expression patterns were also tested for associations with preclinical and clinical parameters, namely, sex, age, French-Amercian-British (FAB) type, white blood cell count (WBC), blast percentage, lactate dehydrogenase (LDH) levels, cytogenetic risk, and achievement of complete remission (CR). At an FDR of <5%, significant associations were found between miRNA levels and blast percentage, cytogenetic risk, and sex (Additional file 4: Table S4).

### Myeloid leukemia cell lines and primary AML samples display a limited pri-miR-221/222 processing capacity

**Figure 1:**
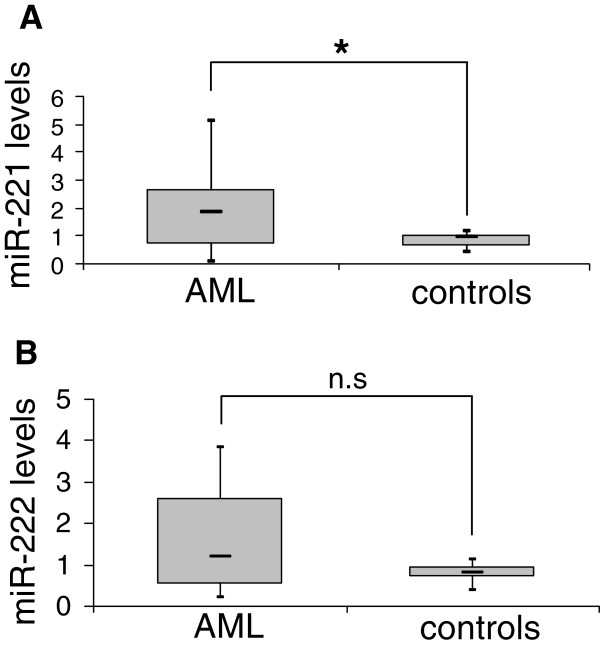
**Mature miR-221 is overexpressed in AML.** 22 blast-enriched AML samples and 9 healthy controls (3 CD34+, 3 BM, 3 PB), all of which had also been included in the microarray experiments, were subjected to Taqman qRT-PCR to measure mature miR-221 **(A)** or miR-222 **(B)**. Expression values were calculated according to the ΔΔCt method [40], with RNU6B as internal reference and PB from healthy donor A as calibrator. *, p < 0.05; n.s., not significant (miR-222, p = 0.064).

miR-221 and miR-222, which together form a cluster located in chromosome band Xp11.3, were chosen for further analyses because i) according to our array data they were among the most strongly overexpressed miRNAs in AML (Additional file 3: Table S3); ii) they are two of only a few miRNAs consistently reported to be overexpressed in AML [12,24,25,44]; and iii) they had been implicated as oncogenes in a number of other malignancies [38,45-49]. qRT-PCR confirmed that mature miR-221 was significantly upregulated in AML compared to healthy controls (Figure 1A). For miR-222, a trend towards differential expression was observed (Figure 1B).

To study the biological effects of miR-221 and miR-222 in human myeloid cells, we introduced suitable expression vectors into the cell lines HL60, KG1, and KG1a. However, neither blasticidin resistant HL60 clones obtained after transfection with miR-Vec-221/222 [38], nor GFP positive HL60, KG1, or KG1a cell populations sorted after infection with pEZX-MR03-miR-221 exhibited increased expression of mature miR-221 or miR-222 (Additional file 5: Figure S1A, and data not shown). In contrast, miR-221 was clearly elevated after transient transfection of HeLa cells with pEZX-MR03-miR-221 (Additional file 5: Figure S1B), demonstrating that this vector was functional. Furthermore, qPCR showed that, other than the mature miR-221, its precursor was indeed overexpressed in the pEZX-MR03-miR-221 infected myeloid cell lines (Additional file 5: Figure S1C), indicating that these cell lines are able to transcribe pre-miR-221 from the pEZX-MR03 vector, but process it inefficiently, possibly due to a saturation effect.

**Figure 2:**
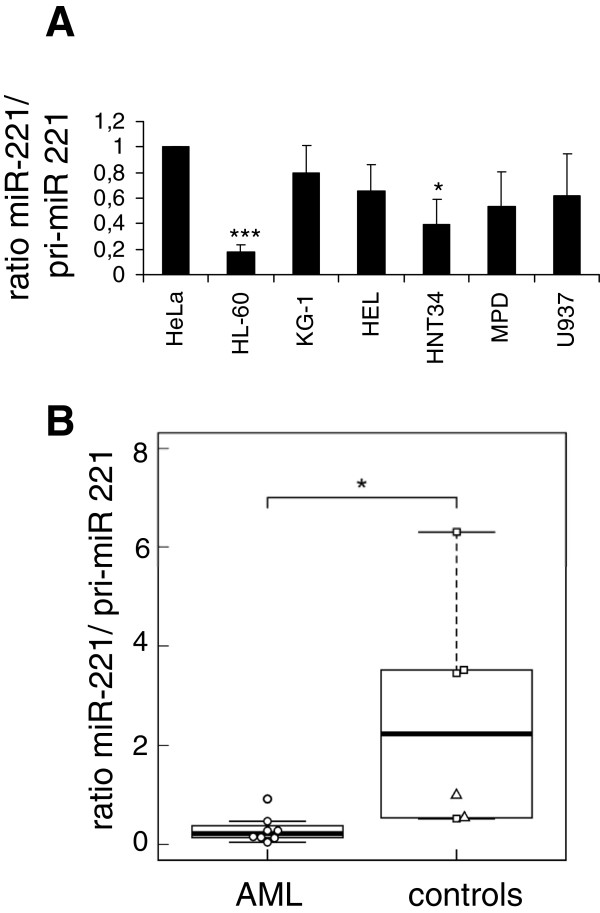
**Malignant human hematopoietic cells display impaired pri-miR-221/222 processing capacity.** Mature miR-221 was measured by qRT-PCR, normalized to RNU6B, and expressed relative to ß-2-microglobulin-normalized pri-miR-221/222. **A)** Human myeloid cell lines, and HeLa cells as a reference; **B)** primary AML samples (n = 8; Additional file 1: Table S1B), enriched for blasts through Ficoll gradient purification, and normal control PB (squares; n = 4) and BM (triangles; n = 2) samples (Additional file 1: Table S1A). HeLa cells were used as a calibrator in both experiments. *, p < 0.05; ***, p < 0.001.

To investigate whether the presumed processing deficiency also affected endogenous miR-221/222, qRT-PCR was performed on a panel of human myeloid cell lines. Because PCR cannot selectively measure pre- versus pri-miRNAs, and efficient reverse transcription of the short pre-miRNA molecules into full-length, PCR amplifiable cDNAs is unlikely based on technical considerations, the RNA species expected to be predominantly measured in these assays is pri-miR-221/222. The miR-221:pri-miR-221/222 ratio was moderately decreased in the myeloid cell lines compared to HeLa cells (Figure 2A), indicating that the capacity of myeloid cells to process endogenous pri-miR-221/222 may be compromised to some extent. More importantly, the miR-221:pri-miR-221/222 ratio was strongly reduced in primary AML cells compared to PB and BM cells from healthy donors (Figure 2B). This could indicate that a pri-miR-221/222 processing defect is acquired during leukemogenesis, or that hematopoietic cells have an a priori limited pri-miR-221/222 processing capacity that becomes saturated when this pri-miRNA is transcriptionally upregulated in the context of leukemia.

### pri-miR-221/222 is universally overexpressed in AML

**Figure 3:**
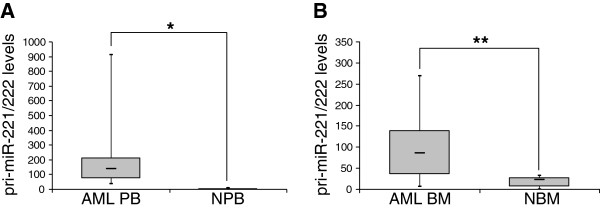
**pri-miR-221/222 is strongly overexpressed in AML.** pri-miR-221/222 levels were measured by qRT-PCR and normalized to those of the housekeeping gene ß-2-microglobulin using the ΔΔct method [40], with PB from healthy donor A as a calibrator in both panels. Please note that the scale of the y-axis differs between the panels. **A)** PB from 18 AML and 6 healthy controls (of which 3 and 4, respectively, had also been used in the experiment shown in Figure 2B; Additional file 1: Tables S1A-C); NPB, normal peripheral blood. **B)** BM from 19 AML (Additional file 1: Table S1C) and 8 healthy controls (Additional file 1: Table S1A; two controls had also been used for the experiment in Figure 2B); NBM, normal bone marrow. AML samples were enriched for leukemic blasts through Ficoll gradient purification. *, p < 0.05; **, p < 0.01.

The experiments shown in Figure 2B had revealed that pri-miR-221/222 was overexpressed to a substantially higher degree than its mature derivatives in AML versus healthy controls. Analysis of additional primary PB samples confirmed that pri-miR-221/222 levels were strongly elevated in AML (mean, 72.4-fold; median, 50.3-fold; range, 13.7 - 332.5-fold relative to the mean expression in 6 healthy PB samples, Figure 3A). Similar results were obtained with BM samples (Figure 3B).

### pri-miRNAs are not generally overexpressed in AML

**Figure 4:**
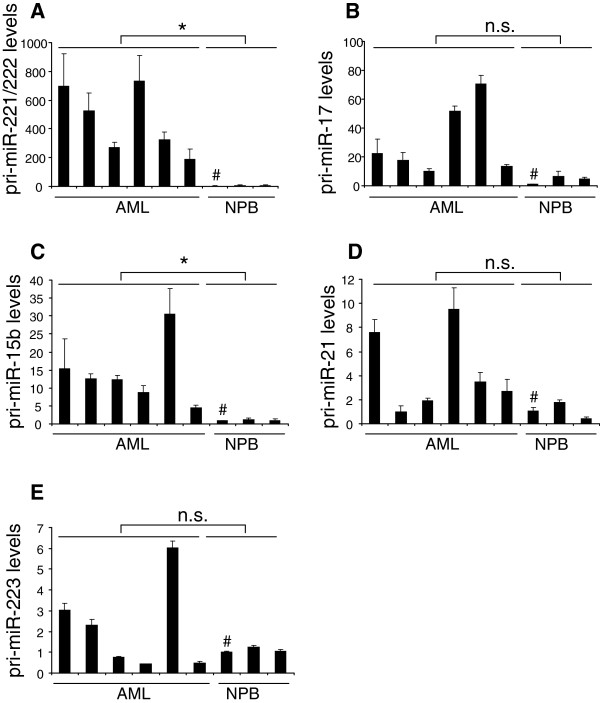
**pri-miRNAs are not generally overexpressed in AML. A)** pri-miR-221/222, **B)** pri-miR-17, **C)** pri-miR-15b, **D)** pri-miR-21, **E)** pri-miR-223. Pri-miRNA levels were measured in 6 blast-enriched AML and 3 normal PB (NPB) samples by qRT-PCR and normalized to those of the housekeeping gene ß-2-microglobulin using the ΔΔct method [40] and PB from donor A (marked with #) as a calibrator. Please note that the scale of the y-axis differs between panels. *, p < 0.05; n.s., not significant.

Since pri-miRNA processing involves both general and miRNA specific factors [17,19-21], the limited processing capacity apparent in AML could be exclusive to pri-miR-221/222 or affect other pri-miRNAs as well. To address this question, 6 AML and 3 normal PB samples were subjected to qRT-PCR for pri-miR-15b, pri-miR-17, pri-miR-21, and pri-miR-223. These pri-miRNAs were chosen because the corresponding mature miRNAs were readily detectable in our microarray analyses, and exhibited different patterns of expression in AML and healthy controls (Additional file 3: Table S3). While mature miR-15b was downregulated in AML (Additional file 3: Table S3), the corresponding pri-miRNA was consistently and significantly overexpressed, albeit to a lesser extent than pri-miR-221/222 (Figure 4A,C). Pri-miR-17, pri-miR-21 and pri-miR-223 were elevated even more moderately and in a non-significant manner (Figure 4B,D,E). These results suggest that AML cells have a limited processing capacity affecting some, but not all, pri-miRNAs, with a particularly strong effect on pri-miR-221/222.

### The pri-miR-221/222 transcript overexpressed in AML is at least 23 kb long

**Figure 5:**
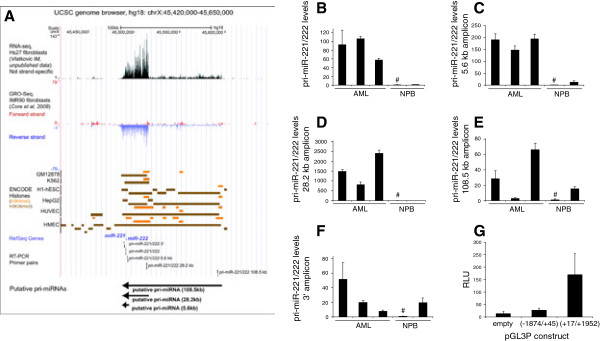
**The pri-miR-221/222 transcript overexpressed in AML is at least 23 kb long. A)** Deep sequencing data and chromatin marks in the relevant region in chromosome band Xp11.3 indicate that 5.6 kb, 28.2 kb, and/or 108.5 kb pri-miR-221/222 transcripts may exist. UCSC genome browser screenshot of chrX: 45,420,000-45,650,000, hg18, showing RNA-seq data from Hs27 foreskin fibroblasts (Vlatkovic IM, unpublished results), GRO-Seq data from IMR90 lung fibroblasts (red, forward, blue, reverse strand) [42], ENCODE histone modification ChIP-seq data for H3K4me3 (orange) and H3K36me3 (brown) in GM12878, K562, H1-hESC, HepG2, HUVEC, and HMEC cells [51], the positions of mature miR-221 and miR-222, the positions of primers used for qRT-PCR, and the putative pri-miR-221/222 transcripts (arrows). B-F) qRT-PCR on 3 blast-enriched AML and 2 healthy control PB samples using primers surrounding mature miR-221 **(B)**, primers near the predicted 5’-ends of the 5.6 kb **(C)**, 28.2 kb **(D)**, and 108.5 kb **(E)** transcripts, and near the 3’ end common to all predicted transcripts **(F)**. Transcript levels were determined by qRT-PCR and normalized to those of the housekeeping gene ß-2-microglobulin using the ΔΔct method [40] and PB from donor A (marked with #) as a calibrator. Note the differences in scale of the y-axis between the different panels. **G)** Luciferase assays demonstrate transcription activating potential of a region near the predicted start site of the 28.2 kb transcript. pGL3-P(-1874/+45), pGL3-P(+17/+1952), and the parental vector pGL3-P were transiently transfected into MCF7 cells, and luciferase activities were determined 2 days later. To control for transfection efficiency, firefly luciferase activity was normalized to renilla luciferase activity expressed from a cotransfected plasmid. Data represent mean +/- SEM from three biological replicates.

The pri-miR-221/222 transcript was previously reported to be ~2.1 kb in size in breast cancer cells [50]. However, publically available global run-on sequencing (GRO-Seq) data from IMR90 primary lung fibroblasts, showing the density of RNA polymerase involved in transcription [42], and RNA-seq data from Hs27 foreskin fibroblasts (IV, manuscript in preparation) indicated the possible existence of pri-miR-221/222 transcripts of 28.2 and 108.5 kb, both expressed from the minus strand (Figure 5A). Their transcriptional orientation was confirmed by ENCODE H3K4me3 peaks, which mark active promoters, and were present in the promoter regions predicted based on the sequencing data. A third putative pri-miR-221/222 transcript of 5.6 kb was mapped based only on H3K4me3 peaks in HMEC cells. All three transcripts would terminate at the same position and contain the sequences for mature miR-221 and miR-222 around 3 and 4 kb, respectively, from their 3’-ends (Figure 5A).

To investigate whether any of these transcripts was expressed in AML, we designed primer pairs for regions near the 5’-ends of each of them, and another pair for a region 3’ of miR-221 (Figure 5A). qRT-PCR on 3 AML and 2 healthy control PB samples showed that the amplicons near the predicted 5’-ends of the 5.6 and 28.2 kb transcripts were strongly elevated in AML, while the amplicon near the 5’-end of the putative 108.5 kb transcript and the 3’ amplicon displayed inconsistent behaviour (Figure 5B-F). Even though these experiments do not formally prove that the amplicons exhibiting similar expression patterns belong to the same transcript, this interpretation is supported by the RNA sequencing data, which strongly suggest the expression of one continuous RNA from this region, and by the fact that according to the UCSC genome browser no other known genes or transcripts are located there. Based on the positions of the primers used (Figure 5A), our results indicate that the pri-miR-221/222 transcript overexpressed in AML starts no more than 2 kb downstream of the 5’-end of the putative 28.2 kb transcript and ends less than 1.5 kb downstream of the miR-221 sequence, making for a total length of at least 23 kb.

To confirm that the region surrounding the presumed pri-miR-221/222 transcriptional start site (TSS) does indeed have transcription activating potential, two fragments encompassing the regions from -1874 to +45 and from +17 to +1952 relative to the estimated start site of the predicted 28.2 kb transcript were cloned into the luciferase reporter vector pGL3-Promoter to yield plasmids pGL3-P(-1874/+45) and pGL3-P(+17/+1952). Because human hematopoietic cell lines are notoriously difficult to transfect, luciferase assays were performed in readily transfectable adherent cell lines. The luciferase activity of pGL3-P(+17/+1952) was approximately 13-fold higher than that of empty pGL3-P in MCF7 cells, while the upstream fragment increased the transcriptional activity of the reporter only about twofold (Figure 5G). Comparable results were obtained in 293 T and HeLa cells (Additional file 6: Figure S2). These data confirm that a region in the immediate vicinity of the predicted pri-miR-221/222 TSS can indeed promote transcription in human cells.

### pri-miR-221/222 levels reflect the disease stage in AML

**Figure 6:**
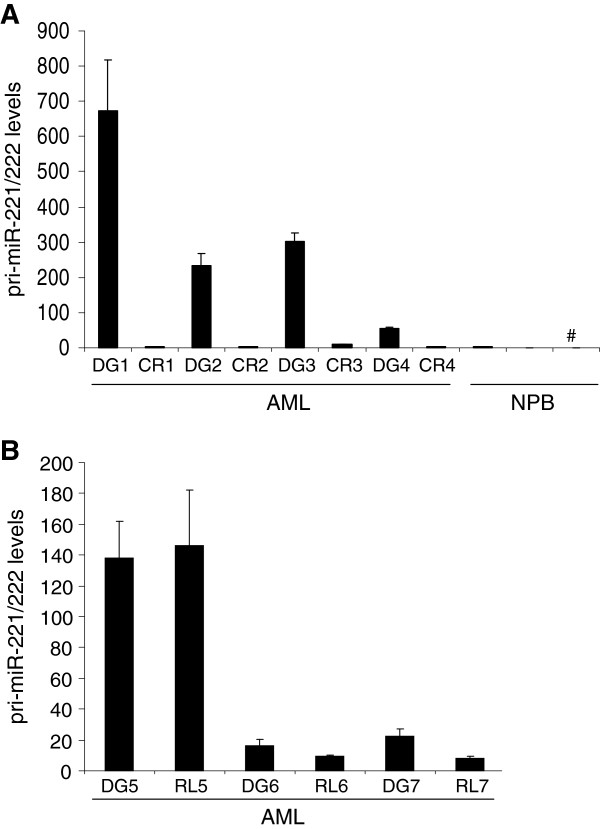
**pri-miR-221/222 levels reflect disease stage in AML. A)** Paired samples from 4 AML patients at the time of diagnosis (DG) and of remission (CR) were compared to 3 healthy control PB (NPB) samples. **B)** Diagnostic (DG) samples from 3 AML patients were compared to paired relapse (RL) samples. Pri-miR-221/222 levels were measured by qRT-PCR and normalized to those of the housekeeping gene ß-2-microglobulin using the ΔΔct method [40] and PB from donor A (marked with # in panel **A)** as a calibrator. Leukemic samples were enriched for blasts using Ficoll.

Finally, we asked whether pri-miR-221/222 expression would reflect the disease stage in AML. qRT-PCR on paired samples from four AML patients from the time of diagnosis and remission showed that pri-miR-221/222 expression was high at the former time point, and declined to control levels at the latter (Figure 6A). In contrast, when paired diagnostic and relapse samples from three AML patients were examined, pri-miR-221/222 expression was comparable between the two time points (Figure 6B). Similar results were obtained with the primers near the 5’-end of the putative 28.2 kb transcript (data not shown).

## Discussion

To identify miRNAs potentially contributing to leukemogenesis, the miRNA expression profiles of 52 AML samples were compared to those of hematopoietic cells from 13 healthy donors. Using microarrays containing highly specific LNA probes for over 600 human miRNAs - a substantially larger number than in most previous studies - 64 miRNAs were found to be misexpressed in AML in a statistically significant manner. Interestingly, subsequent studies on miR-221, one of the miRNAs most consistently overexpressed in AML, revealed a limited capacity of malignant myeloid cells to process its precursor forms. Firstly, only precursor, but not mature miR-221 levels were increased after introduction of appropriate expression vectors into several different human hematopoietic cell lines, and secondly, primary AML cells overexpressed pri-miR-221/222 to a much higher extent than its mature products. Because processing of vector-borne miRNA precursors, like that of endogenous pri-miRNAs, requires consecutive cleavage by Drosha and Dicer, the most plausible explanation of these results is a limited pri-miR-221/222 processing capacity at the Drosha level. pri-miR-221/222 overexpression was present in a high proportion of the patients investigated in this study (84%; 31/37 patients in the experiment shown in Figure 3, using the mean plus 3 standard deviations of the respective healthy controls as a cutoff for overexpression), so that the conclusion appears justified that it is a common phenomenon in AML. Underscoring the association between pri-miR-221/222 overexpression and AML, analysis of samples from the times of diagnosis, remission, and relapse showed that pri-miR-221/222 levels faithfully reflected the stage of disease.

As explanations for the elevated pri-miR-221/222 levels present in AML, pathologically increased transcription on the background of a physiologically limited processing capacity, or an acquired processing deficiency on the background of unaltered transcriptional activity may be considered. In disfavour of the latter possibility, a processing defect at the Drosha level does not necessarily lead to an accumulation of pri-miR molecules [52-56]. Also, it could not easily be reconciled with our own and published [12,24,25,44] observations that mature miR-221 and 222 are not down-, but upregulated in AML. Increased transcription of pri-miR-221/222, in concert with a limited capacity of hematopoietic cells to process this molecule, therefore likely accounts for its induction in AML. This has not been proven, however, and will be the subject of future investigations.

A role for restricted miRNA processing capacities in tumor cells as well as in developmentally immature cells has been previously suggested by Thomson et al. [53]. These authors found that mature let-7 g was almost undetectable in embryonic stem (ES) cells and P19 teratocarinoma cells, although the corresponding pri-miRNA was as abundant as in E14.5 embryos and differentiated P19 cells, which expressed high levels of let-7 g. Similarly, decreased expression of 22 miRNAs in a panel of human tumors of various origins was not accompanied by a reduction in the corresponding pri-miRNAs, indicating that it was due to an acquired processing defect rather than to transcriptional downregulation [53]. In contrast, our data show that in AML transcriptional upregulation on the background of a probably a priori limited processing capacity causes an elevation of pri-miR-221/222 levels. Despite of these differences, both studies concur to suggest a role for restricted miRNA processing in tumorigenesis.

Since pri-miR-221/222 is upregulated in a large majority of AML, either this overexpression per se or an event leading to it should be essential for leukemogenesis. In principle, elevation of pri-miR-221/222 levels could be a bystander effect of the induction of a nearby oncogene. However, according to human genome assembly 19 (http://genome.ucsc.edu/) no known genes are located within a genomic region that extends from 0.7 Mb centromeric to 0.5 Mb telomeric of the mature miR-221 sequence. Also, co-activation of pri-miR-221/222 with an adjacent oncogene would not be able to explain why other pri-miRNAs are also overexpressed in AML. Alternatively, a transcription factor whose deregulation contributes to misexpression of cancer genes could also activate pri-miR-221/222 and other pri-miRNAs in trans. The possible identity of such a transcription factor is presently obscure. Thirdly, an acquired pri-miRNA processing deficiency might contribute to leukemogenesis by diminishing the production of mature miRNAs. This appears unlikely for the reasons outlined above. Fourth, inhibition of pri-miRNA processing could be a side effect of mutations whose primary oncogenic effect is unrelated to miRNA biogenesis. For example, mutations inactivating the transcriptional competence of p53 also interfered with miRNA processing [57]. However, this defect was not accompanied by increased pri-miRNA levels [57], and furthermore p53 mutations are infrequent in AML [58], so that such a mechanism remains speculative at present.

Thus, even though a number of possibilities can be conceived to explain pri-miR-221/222 overexpression as a bystander effect of other oncogenic events, at present none of these is sufficiently plausible to discredit the possibility that pri-miR-221/222 may itself act as an oncogene. lncRNAs have recently received great attention as possible cancer genes with diverse ways of action, e.g. the regulation of chromatin and transcription [59], and pri-miRNAs may represent a novel type of lncRNA. In a recent study, the primary transcripts of the let-7 family of miRNAs (pri-let-7) were reported to be able to regulate gene expression [22], thus ascribing a biochemical function to this type of molecule. Furthermore, some lncRNAs have been reported to serve as precursors for small RNA species in addition to their well described functions as macro ncRNAs [23,60]. Of particular interest, H19, an imprinted RNA that is overexpressed in many tumors and displays oncogenic functions [61,62], has been found to host a miRNA [63]. It can therefore be considered as an example of a pri-miRNA that at the same time acts as an oncogenic lncRNA.

## Conclusion

In this study we identified 64 miRNAs that were differentially expressed between AML and healthy controls, and may therefore contribute to leukemogenesis. Moreover, we found that pri-miR-221/222 was strongly overexpressed, but inefficiently processed, in a large proportion of AML, indicating that it may represent a novel molecular marker and a putative oncogene in this disease.

## Abbreviations

AML: Acute myeloid leukemia; BM: Bone marrow; CR: Complete remission; hg: Human genome assembly; FAB classification: French American British classification; FBS: Fetal bovine serum; FDR: False discovery rate; GRO-Seq: Global run-on sequencing; HSPC: Hematopoietic stem and progenitor cell; LDH: Lactate dehydrogenase; LNA: Locked nucleic acid; lncRNA: Long noncoding RNA; miRNA: MicroRNA; MNC: Mononuclear cells; NBM: Normal bone marrow; NPB: Normal peripheral blood; nt: Nucleotide; PB: Peripheral blood; pre-miRNA: Precursor microRNA; pri-miRNA: Primary microRNA; qRT-PCR: Quantitative reverse transcriptase polymerase chain reaction; RISC: RNA induced silencing complex; SD: Standard deviation; SEM: Standard error of the mean; TSS: Transcriptional start site; UCSC: University of California Santa Cruz; WBC: White blood cell count.

## Competing interests

The authors declare that they have no competing interests.

## Authors’ contributions

AR, KS, CS, ME, MS, and IV designed and performed experiments and interpreted their results. HH carried out statistical and bioinformatic analyses. RK, SCR, PV, and HS provided patient samples and clinical information. RW designed the study, interpreted data, and wrote the manuscript. All other authors read and approved the final manuscript.

## Pre-publication history

The pre-publication history for this paper can be accessed here:

http://www.biomedcentral.com/1471-2407/13/364/prepub

## Supplementary Material

Additional file 1: Table S1 Patient and control samples used in this study. **A)** Healthy controls; **B)** AML samples used for miRNA microarray analyses and qRT-PCR; **C)** additional AML samples used for qRT-PCR; **D)** AML patients with follow-up samples. Cytogenetic risk categories for AML were defined as follows: t(8;21), inv(16), t(15;17), favorable; normal karyotype, other abnormalities, intermediate; 3q21q26 abnormalities, 5q-/-5, 7q-/-7, 11q23 abnormalities, 12p abnormalities, 17p abnormalities, complex aberrant karyotypes (greater than or equal to 3 abnormalities, excluding cases with t(8;21), inv(16), and t(15;17)), unfavorable. Click here for file

Additional file 2: Table S2 **A)** Primers used for Sybr Green based qRT-PCR. Fwd, forward, rev, reverse. Unless specifically mentioned otherwise, pri-miR-221/222 was measured with the primers pri-miR-221/222 fwd and rev, which partially overlap with the miR-221 precursor sequence. **B)** Primers used to amplify and clone the promoter of the putative 28.2 kb pri-miR-221/222 transcript. Fwd, forward, rev, reverse. The transcription initiation site as predicted based on deep sequencing and histone mark data was defined as position +1. The SacI and XhoI restriction sites added for the purpose of cloning are underlined. Click here for file

Additional file 3: Table S3 miRNAs significantly differentially expressed between AML and control samples according to microarray analyses. Separate comparisons were performed between AML and the three types of controls (CD34+; normal BM, NBM; and normal PB, NPB). Only miRNAs that were expressed in at least half of the relevant samples were considered for each comparison. Correction for multiple hypothesis testing was performed according to Benjamini and Hochberg [43]. False discovery rates (FDRs) <0.05 are highlighted in grey. log2 ratios between mean expression values in AML and controls are also indicated; positive values indicate that a miRNA is expressed at higher levels in AML than in the respective control. Click here for file

Additional file 4: Table S4 miRNAs associated with preclinical or clinical parameters in AML. Associations between miRNA levels and sex, age, FAB type, white blood cell count, blast percentage, lactate dehydrogenase (LDH) levels, cytogenetic risk, and achievement of complete remission (CR) were determined as described in Methods. rho, Spearman’s rank correlation coefficient; pbsr, point biserial correlation coefficient; FDR, false discovery rate according to Benjamini and Hochberg [43]. Click here for file

Additional file 5: Figure S1 Human hematopoietic cell lines, but not HeLa cells, fail to process vector borne miR-221. **A)** Human myeloid cell lines HL60, KG1, and KG1a were infected with pEZX-MR03-miR-221 or scrambled control vector and sorted for GFP positivity. Levels of mature miR-221 were determined by Taqman qRT-PCR and normalized to those of RNU6B using the ΔΔct method [40]. For each cell line, non-infected cells were used as a calibrator (not shown). **B)** HeLa cells were transiently transfected with pEZX-MR03-miR-221 or scrambled control vector. 2 days later, miR-221 expression was measured as in A. **C)** GFP positive, pEZX-MR03-miR-221 or control infected HL60, KG1, and KG1a cells were subjected to qRT-PCR for the vector borne precursor form of miR-221. Expression levels were normalized to those of ß-2-microglobulin, using non-infected cells as a calibrator (not shown). Click here for file

Additional file 6: Figure S2 Promoter activity of a region near the predicted start site of the 28.2 kb pri-miR-221/222 transcript. The parental vector pGL3-P and its derivatives pGL3-P(-1874/+45) and pGL3-P(+17/+1952) were transiently transfected into 293 T **(A)** or HeLa **(B)** cells, and luciferase activities were determined 2 days later. To control for transfection efficiency, firefly luciferase activity was normalized to renilla luciferase activity expressed from a cotransfected plasmid. Click here for file
