# Supplementary material for: Overexpression of primary microRNA 221/222 in acute myeloid leukemia
Source: BMC Cancer. 2013 Jul 29;13:364. doi: 10.1186/1471-2407-13-364 (PMC3733744; doi:10.1186/1471-2407-13-364)
Supplement: Additional file 1: Table S1 — Patient and control samples used in this study. A) Healthy controls; B) AML samples used for miRNA microarray analyses and qRT-PCR; C) additional AML samples used for qRT-PCR; D) AML patients with follow-up samples. Cytogenetic risk categories for AML were defined as follows: t(8;21), inv(16), t(15;17), favorable; normal karyotype, other abnormalities, intermediate; 3q21q26 abnormalities, 5q-/-5, 7q-/-7, 11q23 abnormalities, 12p abnormalities, 17p abnormalities, complex aberrant karyotypes (greater than or equal to 3 abnormalities, excluding cases with t(8;21), inv(16), and t(15;17)), unfavorable. [file 1471-2407-13-364-S1.doc]

Additional file 1: Table S1: Patient and control samples used in this study.

A) Healthy controls; B) AML samples used for miRNA microarray analyses and qRT-PCR; C) additional AML samples used for qRT-PCR; D) AML patients with follow-up samples.

Cytogenetic risk categories for AML were defined as follows: t(8;21), inv(16), t(15;17), favorable; normal karyotype, other abnormalities, intermediate; 3q21q26 abnormalities, 5q-/-5, 7q-/-7, 11q23 abnormalities, 12p abnormalities, 17p abnormalities, complex aberrant karyotypes (greater than or equal to 3 abnormalities, excluding cases with t(8;21), inv(16), and t(15;17)), unfavorable [1].

Additional file 1: **Table S1A: Healthy controls, n=21**

**Age**, median (range) 44 (20-57)

**Sex**

Male 11

Female 10

**Tissue**

PB 9

BM 9

BM CD34+ cells 3

Additional file 1: **Table S1B: Diagnostic AML samples used for miRNA microarray analyses and qRT-PCR, n=52**

**Age**, median (range) 64,5 (19-87)

**Sex**

Male 29

Female 23

**Tissue**

PB 52

**Cytogenetic Risk**

Favorable 5

Intermediate 27

Unfavorable 12

Not classsified 8

**FAB type**

M0 4

M1 20

M2 3

M3 1

M4 7

M5 2

secondary AML 14

t-AML 1

Additional file 1: **Table S1C: Additional diagnostic AML samples used for qRT-PCR,** **n=27**

**Age**, median (range) 61 (16-84)

**Sex**

Male 13

Female 14

**Tissue**

PB 6

BM 21

**Cytogenetic Risk**

Favorable 5

Intermediate 14

Unfavorable 5

Not classified 3

**FAB type**

M0 2

M1 3

M2 2

M3 3

M4 7

M5 1

M6 1

biphenotypic AL 1

Not classified 7

Additional file 1: **Table S1D: AML patients with follow-up samples, n=7**

**Age at diagnosis**, median (range) 66 (49-74)

**Sex**

Male 3

Female 4

**Cytogenetic risk**

Favorable 1

Intermediate 2

Unfavorable 2

Not classified 2

**FAB type**

M0/M1 3

M1 1

M3 1

M6 1

Not classified 1

**Diagnostic samples, tissue**

PB 6

BM 1

**CR samples, tissue**

PB 2

BM 2

**Relapse samples, tissue**

PB 3

1. Schoch C, Kern W, Schnittger S, Hiddemann W, Haferlach T: **Karyotype is an independent prognostic parameter in therapy-related acute myeloid leukemia (t-AML): an analysis of 93 patients with t-AML in comparison to 1091 patients with de novo AML.** *Leukemia* 2004, **18**(1):120-12**5.**
