# Supplementary material for: Overexpression of primary microRNA 221/222 in acute myeloid leukemia
Source: BMC Cancer. 2013 Jul 29;13:364. doi: 10.1186/1471-2407-13-364 (PMC3733744; doi:10.1186/1471-2407-13-364)
Supplement: Additional file 2: Table S2 — A) Primers used for Sybr Green based qRT-PCR. Fwd, forward, rev, reverse. Unless specifically mentioned otherwise, pri-miR-221/222 was measured with the primers pri-miR-221/222 fwd and rev, which partially overlap with the miR-221 precursor sequence. B) Primers used to amplify and clone the promoter of the putative 28.2 kb pri-miR-221/222 transcript. Fwd, forward, rev, reverse. The transcription initiation site as predicted based on deep sequencing and histone mark data was defined as position +1. The SacI and XhoI restriction sites added for the purpose of cloning are underlined. [file 1471-2407-13-364-S2.doc]

Additional file 2: Table S2A: Primers used for Sybr Green based qRT-PCR.

Fwd, forward, rev, reverse. Unless specifically mentioned otherwise, pri-miR-221/222 was measured with the primers pri-miR-221/222 fwd and rev, which partially overlap with the miR-221 precursor sequence.

| **Primer Name** | **Primer sequence (5’ -> 3’)** |
| --- | --- |
| beta-2-microglobulin fwd | ATGAGTATGCCTGCCGTGTGA |
| beta-2-microglobulin rev | GGCATCTTCAAACCTCCATG |
| pri-miR-221/222 fwd | ACTTGCAAGCTGAACATCCA |
| pri-miR-221/222 rev | CCTGAAACCCAGCAGACAAT |
| pri-miR-223 fwd | AAGCACGATTTGGGAAACTG |
| pri-miR-223 rev | TTCTTAGGCAGATGGCTGGT |
| pri-miR-21 fwd | TGGGGTTCGATCTTAACAGG |
| pri-miR-21 rev | TGTCACGATGGTAGGCAAAA |
| pri-miR-17 fwd | CCAGACTTTGGCAACAGTGA |
| pri-miR-17 rev | GGCAGAAATGCTGGGTAAAA |
| pri-miR-15b fwd | CGGTCCCGTTGTAAGTCTGT |
| pri-miR-15b rev | CTACCCCTGAATGGAGCAAA |
| pri-miR-221/222 3’ fwd | GGTGCCAGGTGTGAAGAACT |
| pri-miR-221/222 3’ rev | GCTTTGAGGATCAACCCAAT |
| pri-miR-221/222 5.6 kb fwd | AGAGCTCCCAGAAAGCTGGT |
| pri-miR-221/222 5.6 kb rev | TTTGATCGGACATGCAGCTA |
| pri-miR-221/222 28.2 kb fwd | ACTTGCCCTCCTTTCCTTTC |
| pri-miR-221/222 28.2 kb rev | AGGTGTTTCCGACGCATTAC |
| pri-miR-221/222 108.2 kb fwd | AAGGGTCAGGCATCAAAATG |
| pri-miR-221/222 108.2 kb rev | TCGAGGTAACACCACAACCA |

Additional file 2: Table S2B: Primers used to amplify and clone the promoter of the putative 28.2 kb pri-miR-221/222 transcript.

Fwd, forward, rev, reverse. The transcription initiation site as predicted based on deep sequencing and histone mark data was defined as position +1. The SacI and XhoI restriction sites added for the purpose of cloning are underlined.

| **Primer Name** | **Primer sequence (5’ -> 3’)** |
| --- | --- |

| P-pri-miR-221/222(-1874/+45) fwd | AGCGAGCTCGATTCTGCACTGGCCTTAGC |
| --- | --- |
| P-pri-miR-221/222(-1874/+45) rev | AGCTCTCGAGTTTTGATCGTGCAAGACTGC |
| P-pri-miR-221/222(+17/+1952) fwd | AGCGAGCTCGGAGCTGTTGCAGTCTTGCAC |
| P-pri-miR-221/222(+17/+1952) rev | AGCTCTCGAGAGGTGTTTCCGACGCATTAC |
