# Supplementary material for: Overexpression of primary microRNA 221/222 in acute myeloid leukemia
Source: BMC Cancer. 2013 Jul 29;13:364. doi: 10.1186/1471-2407-13-364 (PMC3733744; doi:10.1186/1471-2407-13-364)
Supplement: Additional file 3: Table S3 — miRNAs significantly differentially expressed between AML and control samples according to microarray analyses. Separate comparisons were performed between AML and the three types of controls (CD34+; normal BM, NBM; and normal PB, NPB). Only miRNAs that were expressed in at least half of the relevant samples were considered for each comparison. Correction for multiple hypothesis testing was performed according to Benjamini and Hochberg [43]. False discovery rates (FDRs) <0.05 are highlighted in grey. log2 ratios between mean expression values in AML and controls are also indicated; positive values indicate that a miRNA is expressed at higher levels in AML than in the respective control. [file 1471-2407-13-364-S3.doc]

Additional file 3: Table S3: miRNAs significantly differentially expressed between AML and control samples according to microarray analyses.

Separate comparisons were performed between AML and the three types of controls (CD34+; normal BM, NBM; and normal PB, NPB). Only miRNAs that were expressed in at least half of the relevant samples were considered for each comparison. Correction for multiple hypothesis testing was performed according to Benjamini and Hochberg [1]. False discovery rates (FDRs) <0.05 are highlighted in grey. log2 ratios between mean expression values in AML and controls are also indicated; positive values indicate that a miRNA is expressed at higher levels in AML than in the respective control.

|  | AML vs. CD34+ | |  | AML vs. NBM | |  | AML vs. NPB | |
| --- | --- | --- | --- | --- | --- | --- | --- | --- |
| miRNA name | FDR | log2 ratio |  | FDR | log2 ratio |  | FDR | log2 ratio |
| hsa-let-7a | 0.963 | 0.01 |  | 3.5E-05 | 1.34 |  | 0.138 | 0.28 |
| hsa-let-7b | 3.6E-03 | 0.99 |  | 8.2E-04 | 1.65 |  | 1.5E-02 | 0.68 |
| hsa-let-7c | 0.445 | 0.30 |  | 1.1E-02 | 1.40 |  | 3.8E-02 | 0.60 |
| hsa-let-7g | 0.879 | 0.20 |  | 0.691 | -0.18 |  | 2.9E-02 | -1.34 |
| hsa-miR-15a | 0.423 | 1.51 |  | 9.2E-03 | 0.38 |  | 0.120 | -0.30 |
| hsa-miR-15b | 0.289 | -0.66 |  | 0.303 | 0.19 |  | 5.0E-03 | -1.13 |
| hsa-miR-16 | 0.695 | 0.18 |  | 3.4E-03 | 0.39 |  | 2.0E-03 | -0.79 |
| hsa-miR-17 | 1.1E-02 | -0.91 |  | 2.8E-06 | 1.45 |  | 4.6E-15 | 1.24 |
| hsa-miR-19a | 6.2E-03 | 0.55 |  | 0.348 | 0.26 |  |  |  |
| hsa-miR-19b | 3.6E-03 | -0.82 |  | 8.1E-09 | 1.28 |  | 1.3E-04 | 1.11 |
| hsa-miR-20a | 0.501 | -0.18 |  | 1.5E-09 | 1.50 |  | 1.3E-10 | 1.58 |
| hsa-miR-21 | 0.084 | 4.02 |  | 1.4E-02 | 0.96 |  | 0.113 | 0.41 |
| hsa-miR-23a | 0.084 | 0.39 |  | 1.1E-04 | 0.81 |  | 1.6E-05 | -0.90 |
| hsa-miR-23b | 0.119 | 0.26 |  | 9.3E-04 | 0.87 |  | 1.5E-07 | -1.25 |
| hsa-miR-24 | 0.362 | 0.19 |  | 4.0E-03 | 1.01 |  | 1.9E-03 | -1.16 |
| hsa-miR-26a | 0.688 | -0.15 |  | 0.443 | -0.13 |  | 1.3E-04 | -1.64 |
| hsa-miR-27a | 0.096 | 1.92 |  | 1.5E-04 | 1.37 |  | 0.446 | -0.14 |
| hsa-miR-27b | 0.273 | 1.94 |  | 1.8E-02 | 1.29 |  | 0.514 | -0.15 |
| hsa-miR-29a | 0.332 | 1.63 |  | 1.8E-02 | -0.81 |  | 3.5E-03 | -2.18 |
| hsa-miR-30a | 0.501 | 0.75 |  | 6.9E-05 | 0.35 |  | 0.115 | -0.38 |
| hsa-miR-30b | 0.240 | -0.49 |  | 2.9E-04 | 0.93 |  | 0.164 | -0.39 |
| hsa-miR-30b* |  |  |  | 1.2E-03 | -0.63 |  | 0.585 | -0.28 |
| hsa-miR-30c | 0.119 | -0.50 |  | 0.063 | 0.67 |  | 1.2E-03 | -0.99 |
| hsa-miR-92a | 0.096 | -0.92 |  | 1.8E-05 | 1.76 |  | 2.1E-03 | 0.87 |
| hsa-miR-101 | 0.839 | 0.16 |  | 1.1E-02 | 0.25 |  | 0.148 | -0.51 |
| hsa-miR-103 |  |  |  | 0.243 | -0.26 |  | 1.3E-08 | -1.26 |
| hsa-miR-106a | 3.6E-03 | -0.70 |  | 4.7E-11 | 1.57 |  | 1.8E-12 | 1.28 |
| hsa-miR-106b | 2.8E-02 | -0.44 |  | 0.402 | 0.19 |  | 0.239 | -0.26 |
| hsa-miR-107 | 0.501 | 0.35 |  | 0.605 | 0.18 |  | 1.6E-11 | -1.38 |
| hsa-miR-126 | 2.2E-02 | -2.14 |  | 3.4E-07 | 2.12 |  | 0.183 | -0.56 |
| hsa-miR-191 | 0.788 | -0.15 |  | 2.0E-04 | 0.96 |  | 3.1E-03 | -1.14 |
| hsa-miR-193a-5p |  |  |  | 0.514 | -0.12 |  | 3.0E-05 | 0.93 |
| hsa-miR-210 | 0.253 | 1.04 |  | 2.6E-06 | -1.43 |  | 4.1E-03 | 1.78 |
| hsa-miR-212 | 0.677 | -0.35 |  | 0.938 | -0.01 |  | 1.9E-03 | -0.70 |
| hsa-miR-22 | 0.232 | 2.57 |  |  |  |  | 3.9E-09 | -2.51 |
| hsa-miR-221 | 0.839 | 0.19 |  | 8.6E-05 | 2.59 |  | 1.3E-04 | 1.69 |
| hsa-miR-222 | 0.754 | -0.11 |  |  |  |  | 1.1E-03 | 1.68 |
| hsa-miR-223 | 3.7E-02 | 0.77 |  | 0.124 | -0.39 |  |  |  |
| hsa-miR-296-5p | 0.431 | 0.56 |  | 2.7E-07 | -0.90 |  | 0.338 | 0.67 |
| hsa-miR-326 |  |  |  | 4.7E-06 | -1.54 |  |  |  |
| hsa-miR-340 | 1.3E-02 | -0.54 |  | 9.5E-04 | 0.28 |  | 0.138 | 0.43 |
| hsa-miR-423-3p |  |  |  | 1.6E-10 | -1.76 |  | 0.370 | 0.21 |
| hsa-miR-483-3p | 0.170 | -1.06 |  | 1.2E-08 | -1.17 |  | 0.138 | 0.52 |
| hsa-miR-487b | 0.595 | 0.24 |  | 0.487 | -0.20 |  | 6.2E-03 | -0.89 |
| hsa-miR-491-3p | 0.980 | -0.01 |  | 5.0E-04 | -1.76 |  | 1.6E-02 | -0.73 |
| hsa-miR-498 | 0.492 | 0.10 |  | 1.2E-02 | -0.57 |  | 0.091 | 0.37 |
| hsa-miR-503 | 0.728 | 0.21 |  | 3.5E-05 | -1.04 |  | 2.1E-03 | 2.04 |
| hsa-miR-519d | 0.431 | -0.63 |  | 2.2E-02 | -0.38 |  | 1.1E-02 | -0.68 |
| hsa-miR-525-5p | 0.705 | -0.26 |  | 2.2E-02 | -0.35 |  | 0.241 | 0.40 |
| hsa-miR-665 | 0.107 | -0.40 |  | 3.5E-05 | -1.23 |  | 0.370 | -0.54 |
| hsa-miR-668 | 6.2E-03 | -1.38 |  | 0.081 | 0.45 |  | 0.673 | -0.08 |
| hsa-miR-671-5p |  |  |  | 8.1E-03 | -0.54 |  | 0.511 | -0.25 |
| hsa-miR-765 | 6.2E-03 | -1.13 |  | 5.9E-05 | -1.17 |  | 0.653 | 0.25 |
| hsa-miR-766 | 0.501 | -0.42 |  | 9.3E-07 | -2.71 |  | 4.7E-02 | 0.90 |
| hsa-miR-768-3p | 0.790 | -0.08 |  | 0.443 | 0.22 |  | 1.5E-02 | -1.15 |
| hsa-miR-768-5p | 0.177 | -0.83 |  |  |  |  | 1.9E-02 | -1.36 |
| hsa-miR-801 | 1.2E-05 | -1.07 |  | 0.515 | 0.14 |  | 0.141 | 0.65 |
| miRPlus_17869 | 0.084 | -1.24 |  | 0.477 | 0.10 |  | 2.1E-05 | 0.49 |
| miRPlus_17890 | 0.334 | 0.55 |  | 8.3E-04 | -0.62 |  | 0.585 | -0.16 |
| miRPlus_17952 | 0.096 | -1.36 |  | 4.7E-11 | -2.64 |  | 0.941 | -0.02 |
| miRPlus_21472 | 0.839 | 0.10 |  | 3.8E-09 | -1.54 |  | 2.7E-02 | 0.63 |
| miRPlus_27561 | 0.714 | 0.27 |  | 1.9E-05 | -1.53 |  | 5.0E-03 | 1.33 |
| miRPlus_27564 | 6.2E-03 | -0.72 |  | 2.8E-06 | -1.13 |  | 0.456 | -0.31 |
| miRPlus_28232 | 0.119 | -0.55 |  | 4.7E-11 | -1.15 |  | 1.8E-02 | 0.86 |

1. Benjamini Y, Hochberg Y: **Controlling the false discovery rate: a practical and powerful approach to multiple testing.** *J R Statist Soc B* 1995, **57**(1):289-300.
