# Supplementary material for: Overexpression of primary microRNA 221/222 in acute myeloid leukemia
Source: BMC Cancer. 2013 Jul 29;13:364. doi: 10.1186/1471-2407-13-364 (PMC3733744; doi:10.1186/1471-2407-13-364)
Supplement: Additional file 4: Table S4 — miRNAs associated with preclinical or clinical parameters in AML. Associations between miRNA levels and sex, age, FAB type, white blood cell count, blast percentage, lactate dehydrogenase (LDH) levels, cytogenetic risk, and achievement of complete remission (CR) were determined as described in Methods. rho, Spearman’s rank correlation coefficient; pbsr, point biserial correlation coefficient; FDR, false discovery rate according to Benjamini and Hochberg [43]. [file 1471-2407-13-364-S4.doc]

Additional file 4: Table S4: miRNAs associated with preclinical or clinical parameters in AML.

Associations between miRNA levels and sex, age, FAB type, white blood cell count, blast percentage, lactate dehydrogenase (LDH) levels, cytogenetic risk, and achievement of complete remission (CR) were determined as described in Methods.

rho, Spearman's rank correlation coefficient; pbsr, point biserial correlation coefficient; FDR, false discovery rate according to Benjamini and Hochberg [1].

| **Associations** | | **strength (rho, pbsr)** | **FDR** |
| --- | --- | --- | --- |
| blast percentage | hsa-miR-665 | -0.489 | 0.014 |
|  | miRPlus_27564 | -0.478 | 0.014 |
|  | hsa-miR-32* | -0.458 | 0.014 |
| Cytogenetic risk | hsa-miR-101 | 0.494 | 0.036 |
|  | hsa-miR-17 | 0.503 | 0.030 |
|  | hsa-miR-19b | 0.468 | 0.036 |
| Sex^#^ | miRPlus_17869 | 0.436 | 0.040 |
|  |  |  |  |
|  | ^#^miRPlus_17869 levels are higher in females than in males |  |  |

1. Benjamini Y, Hochberg Y: **Controlling the false discovery rate: a practical and powerful approach to multiple testing.** *J R Statist Soc B* 1995, **57**(1):289-300.
