# Supplementary figures and images for: Overexpression of primary microRNA 221/222 in acute myeloid leukemia
Source: BMC Cancer. 2013 Jul 29;13:364. doi: 10.1186/1471-2407-13-364 (PMC3733744; doi:10.1186/1471-2407-13-364)

Fig. S1

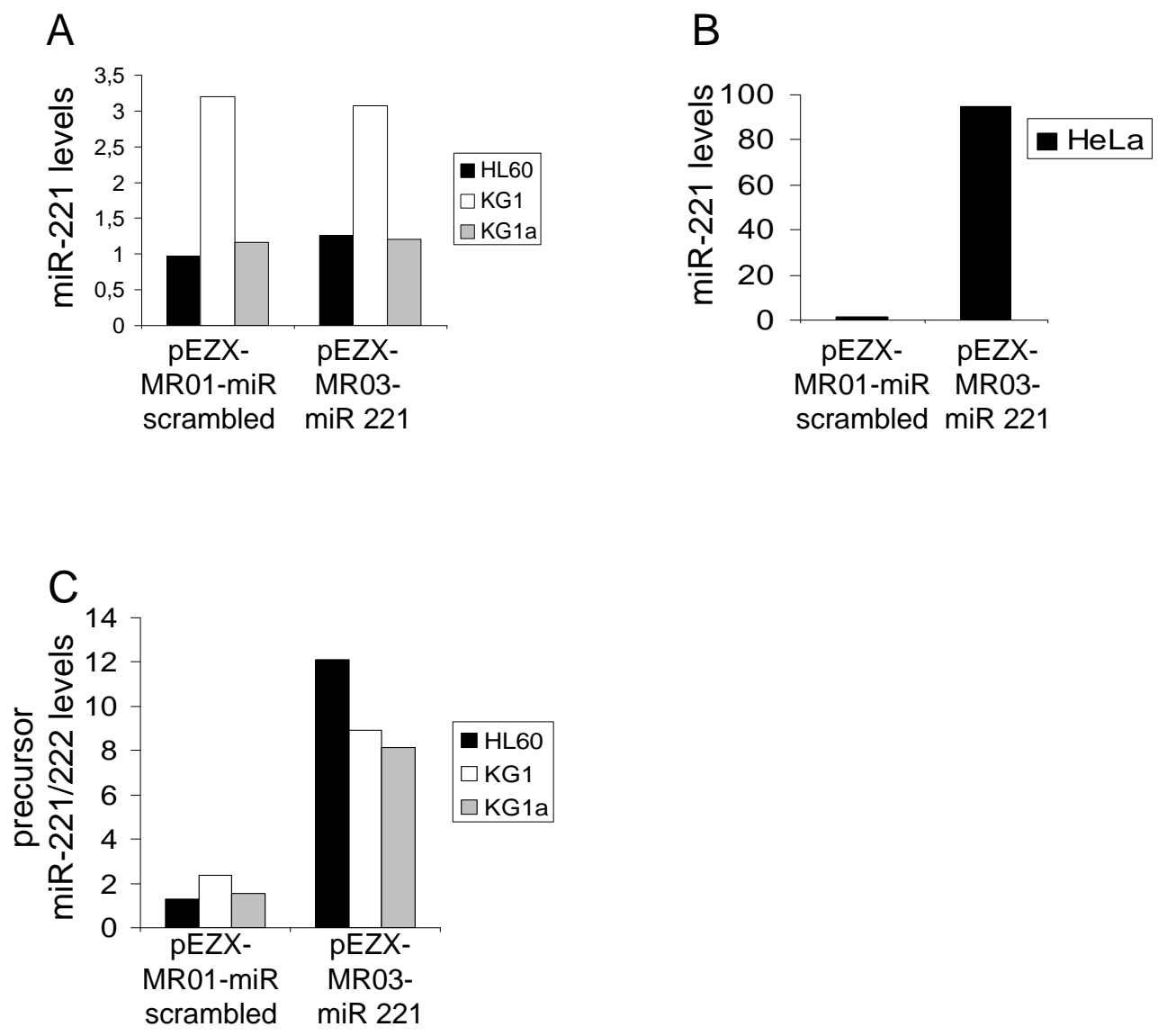

Supplement: Additional file 5: Figure S1 — Human hematopoietic cell lines, but not HeLa cells, fail to process vector borne miR-221. A) Human myeloid cell lines HL60, KG1, and KG1a were infected with pEZX-MR03-miR-221 or scrambled control vector and sorted for GFP positivity. Levels of mature miR-221 were determined by Taqman qRT-PCR and normalized to those of RNU6B using the ΔΔct method [40]. For each cell line, non-infected cells were used as a calibrator (not shown). B) HeLa cells were transiently transfected with pEZX-MR03-miR-221 or scrambled control vector. 2 days later, miR-221 expression was measured as in A. C) GFP positive, pEZX-MR03-miR-221 or control infected HL60, KG1, and KG1a cells were subjected to qRT-PCR for the vector borne precursor form of miR-221. Expression levels were normalized to those of ß-2-microglobulin, using non-infected cells as a calibrator (not shown). [file 1471-2407-13-364-S5.pdf]

Fig. S2

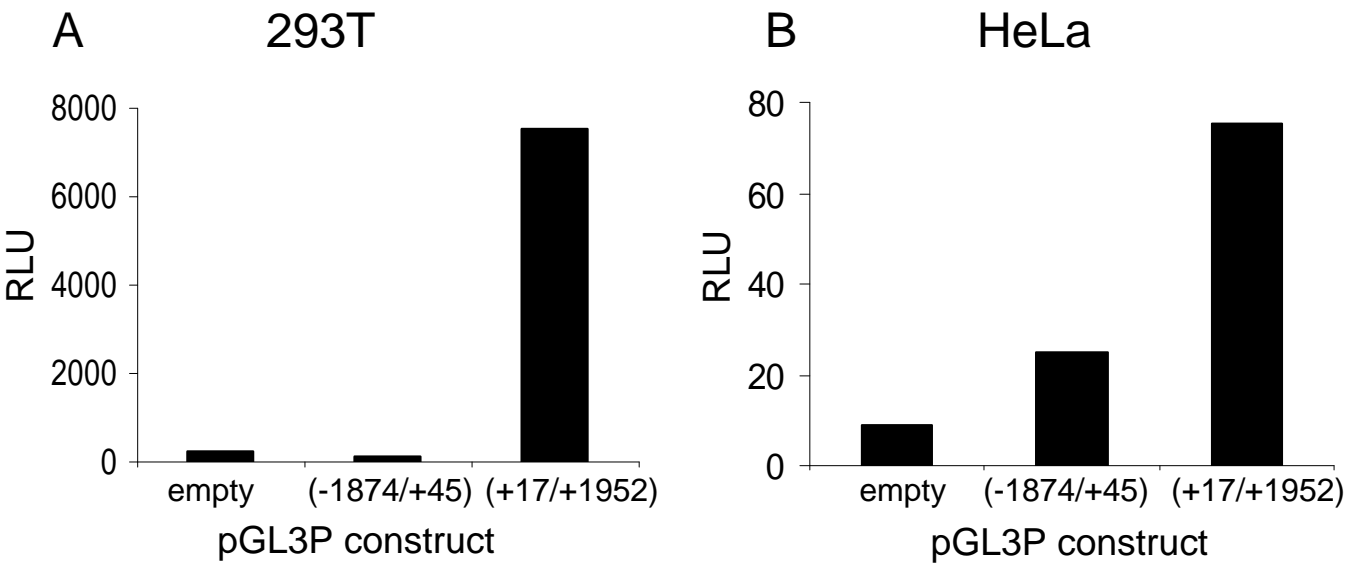

Supplement: Additional file 6: Figure S2 — Promoter activity of a region near the predicted start site of the 28.2 kb pri-miR-221/222 transcript. The parental vector pGL3-P and its derivatives pGL3-P(-1874/+45) and pGL3-P(+17/+1952) were transiently transfected into 293 T (A) or HeLa (B) cells, and luciferase activities were determined 2 days later. To control for transfection efficiency, firefly luciferase activity was normalized to renilla luciferase activity expressed from a cotransfected plasmid. [file 1471-2407-13-364-S6.pdf]
